# Multifractal Framework Based on Blanket Method

**DOI:** 10.1155/2014/894546

**Published:** 2014-01-22

**Authors:** Milorad P. Paskaš, Irini S. Reljin, Branimir D. Reljin

**Affiliations:** ^1^School of Electrical Engineering, University of Belgrade, Bulevar kralja Aleksandra 73, 11121 Belgrade, Serbia; ^2^Innovation Center of School of Electrical Engineering, University of Belgrade, Bulevar kralja Aleksandra 73, 11121 Belgrade, Serbia

## Abstract

This paper proposes two local multifractal measures motivated by blanket method for calculation of fractal dimension. They cover both fractal approaches familiar in image processing. The first two measures (proposed Methods 1 and 3) support model of image with embedded dimension three, while the other supports model of image embedded in space of dimension three (proposed Method 2). While the classical blanket method provides only one value for an image (fractal dimension) multifractal spectrum obtained by any of the proposed measures gives a whole range of dimensional values. This means that proposed multifractal blanket model generalizes classical (monofractal) blanket method and other versions of this monofractal approach implemented locally. Proposed measures are validated on Brodatz image database through texture classification. All proposed methods give similar classification results, while average computation time of Method 3 is substantially longer.

## 1. Introduction

Fractality is often introduced through geometry [1, 2] but it is valid whenever dimensionality is in the focus of research. Both geometry and calculus consider dimension as a point's relation to its neighborhood and the trend of behavior of the geometrical object or function in the surrounding of the point.

After its introduction in image processing [3–5] in 1980s, fractal model has been used in texture classification [6, 7], image segmentation [8–10], image compression [11], and edge detection [12]. Contrary to statistical models of the image, where image is perceived as a union of regions with homogeneity of chosen statistical moment (it is usually mean value of the region), fractality models an image as a union of regions with homogeneity of dimension or spectrum of dimensions. As it will be described in detail in Section 2, dimension is a nonlinear attribute of the object (region). This is of special interest when analyzing textures since the statistical moments of textural regions in an image do not describe them uniquely.

This paper proposes two local multifractal (MF) measures based on blanket method [3] for calculation of fractal dimension. Blanket method is based on monofractal model of an image, where the image is modeled as a three-dimensional surface and, hence, pixel intensity is seen as a third spatial component. The area of this surface—blanket—is assumed to be monofractal. Area is calculated by thickening the blanket and then dividing its volume by thickness of the blanket. Fractal model assumes that area of the surface is growing this way with the power of dimension. While blanket method treats an image as a monofractal object, proposed measures expand blanket method to multifractality. Each of proposed measures represents an example of two directions in implementation of fractality in image processing. Existing techniques developed within one of two approaches exclude the other approach. This paper covers mutually both approaches and employs methods for calculation of local and global dimension from both models.

The paper is organized as follows. Section 2 deals with dimensionality and introduces multifractality in general. Overview of applications of multifractal method in image processing is given in Section 3.  Section 4 gives a brief survey on blanket method which inspired multifractal measures proposed in Section 5. Finally, results and experimental settings are represented in Section 6. Conclusion and further discussion on obtained results are given in Section 7. Further explanation of box-counting method and Legendre MF spectrum is given in the Appendix in order to cover all aspects of multifractality.

## 2. Dimensionality

### 2.1. Reference Model

**Figure 1:**
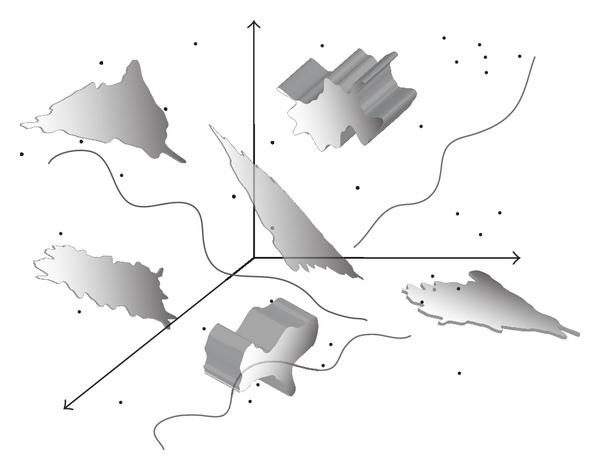
Reference model as a setting for discussion on dimensionality. Intersections of objects of different dimensionality are permitted in this model.

**Figure 2:**
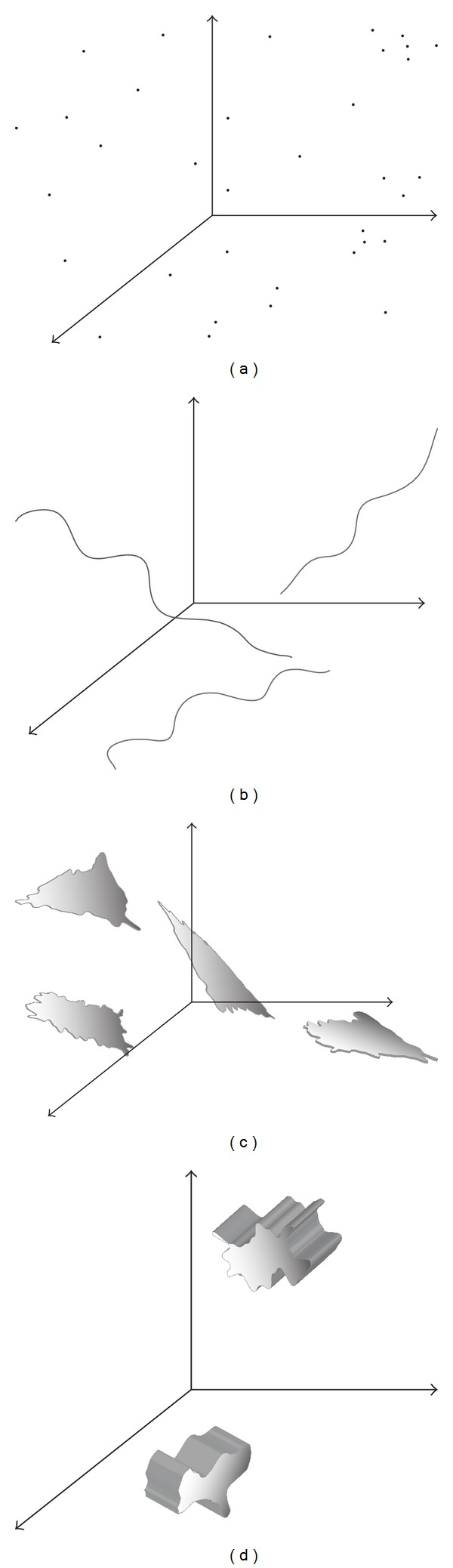
Decomposition of a reference model from Figure 1 on subsets with dimensions 0, 1, 2, and 3.

Dimensionality is usually considered in a sense (interpretation) of geometry. Classical (nonfractal) approach considers an object's *embedded dimension*, that is, dimension of the hyperspace that embodies that object. Furthermore, this model implies only four discrete values of dimension: 0 for point, 1 for curve, 2 for plane, and 3 for solid body. Determination of a dimension of an object is therefore heuristic. In order to become cognizant of dimension determination task, we created simple reference model for our further discussion as shown in Figure 1. Object given in Figure 1 is a mixture of objects with different dimensions and represents a multidimensional object. This object cannot be observed as an object with only one value of dimension (unidimensional) but rather as a multidimensional set of points (or objects) with unidimensional subsets (Figure 2). This is clearer when inspecting differences between points belonging to different subsets. Thus, points from one-dimensional subsets have only one degree of freedom in sense of connectedness with other points from that subset. In other words there is only one way in connecting two distinct points within the one-dimensional object. Concept of connectedness as far as a model of dimensionality is closely related to differentiability of a function (object). It also seeks for trend of behavior [13] of a function in the neighborhood of observed point. Local trend of a function indicates *topological dimension* of a function or an object.

The first step toward dimensional analysis is to isolate subsets with the same dimension or singularity as explained before and then to find its dimension. The quality that discriminates subsets with different dimensions is a *measure* of each subset. It can be number of points within the subset, length, area, or volume. Measure is so-called *set function* since it maps a set of points into one point—measured value. Hence, every subset is fully described either by a measure or a dimension; these two qualities relate to the same property of an object. Significance of measure and measuring becomes apparent with objects with irregular shapes such as surfaces in Figure 1. When calculating area of any of these surfaces it must be divided into small “regular” coverings whose area is straightforwardly (in the same manner) calculated. Total area is then given through summing (integrating) all divided parts. Measure—dimension dualism can be further described as follows:

(1a) $$\begin{array}{c} M\left(F_{i}\right)=\sum_{F_{i}}\delta^{D_{i}}=N_{i}\left(\delta \right)\cdot \delta^{D_{i}}, \end{array}$$

(1b) $$\begin{array}{c} D\left(F_{i}\right)=\frac{\log\left(M/N_{i}\right)}{\log\left(\delta \right)}\sim \frac{\log\left(M\right)}{\log\left(\delta \right)}, \end{array}$$

where *M*(*F*_*i*_) is a measure of a subset *F*_*i*_, *N*_*i*_ is the number of covers, and *D*_*i*_ is the integer-valued dimension of the subset. We will recall scaling manner such as one in (1a) and (1b) many times throughout this paper. Described model of dimensionality reduces measurement on one-dimensional (unit) gauge, *δ*; other dimension values are deduced from the unit gauge. Thus, other order dimensions are determined in comparison to one-dimensional reference. However, this model assumes covering of a subset with identical coverings. This could be excessively strict restriction in some cases.

#### 2.1.1. Digression

In this paper terms *measure* and *capacity* will be used interchangeably. More precisely capacity can be seen as a measure which is not additive [14]. Since mathematically correct explanation is beyond of the scope of this paper and MF formalism holds for capacities, additivity will not be examined.

### 2.2. Unidimensionality (Monofractality)

In mid-nineteenth century mathematicians created geometrical objects with “strange” behavior. One example is the famous Peano curve; it is a curve which fills the plane through infinite number of iterations. Its embedded dimension is surely two, although its topological dimension (dimension of the original curve) is one.

As measure and dimension inquire function integrability (i.e., differentiability) it is not possible to measure or calculate dimension for nondifferentiable points of the function. Indeed there are nondifferentiable functions in all points. An example is Weierstrass function. Therefore, dimensionality should be broadened to wider range of values. Generalization of dimensionality assumes fractional (nonintegral) value of dimension; this value is between integral values of topological and embedded dimension. Objects with non-integral dimensions are called *fractals *[1]. This generalization spreads on fractional derivatives but it is out of scope of this paper.

Now we can imagine set *F* from reference model as a union of fractal subsets (Figure 2). In order to incorporate fractional dimensions into existing model, Hausdorff [15] generalized model in (1a) and (1b):

(2a) $$\begin{array}{c} M_{H}\left(F_{i},s\right)=\underset{\delta \to 0}{\lim}\inf\sum_{\begin{array}{c} j=1\\ F_{i} \end{array}}^{\infty }{\left|U_{j}\right|}^{s}, \end{array}$$

where {*U*_*i*_} is a *δ*-cover of unidimensional subset, *F*_*i*_, and *s* can be fractional. Dimension of a subset *F*_*i*_ is defined correspondingly:

(2b) DH(Fi)=inf⁡{s≥0:MH(Fi,s)=0}=sup⁡{s:MH(Fi,s)=∞}.

This definition of dimension is stringent and it encompasses previous model given in (1a) and (1b) in case of fractional dimensions. Definition in (2b) simply says that there is only one value of *s* for which measure in (2a) is nontrivial. Also it implicitly shows that dimension is defined with respect to measure and it is obvious now that different measures defined on the same set will give different values of dimension.

### 2.3. Multidimensionality (Multifractality)

Hitherto we have been analyzing dimensions of unidimensional subsets, *F*_*i*_, of a multidimensional set *F*. It is based on assumption that we have a priori knowledge of dimensionality of each subset and we only have to calculate its quantity, that is, dimension. This solution is adequate only if the fractal synthesis is known in advance. Unfortunately, there are many situations when this assumption is lacking. Connectedness or function trend is varying on the atomic level, namely, from point to point. It leads to the conclusion that measure should be introduced in different manner than in reference model. This is the main reason for introducing *local dimension* or *Hölder exponent of measure μ* assigned to a point rather than to a set:

(3) $$\begin{array}{c} d_{\mu }\left(x\right)\sim \underset{\delta \to 0}{\lim}\frac{\log\mu \left({ℬ}_{\delta }\left(x\right)\right)}{\log\delta }. \end{array}$$

Local dimension is therefore a modification of global or outer dimension from (1a) and (1b) and (2a) and (2b) since it is calculated on a ball *ℬ*_*δ*_(**x**) centered in **x**, with radius *δ*, and assigned to a single point instead to a set of points. Local dimension is usually in the literature denoted with *α* [2]. Chosen annotation in this paper is suggestive and it points to dependence of local dimension on selected measure.

Determination of local dimension for all points within multidimensional set *F* categorizes points into unidimensional classes, *F*_*i*_, with respect to selected measure, *μ*:

(4a) $$\begin{array}{c} F_{i}=\left\{x\in F:d_{\mu }\left(x\right)=d_{i}\right\}. \end{array}$$

Now it is possible to calculate dimension of each monofractal subset using, for example, (2a) and (2b); instead of Hausdorff measure, dimension can be calculated using any other definition of measure. Furthermore, *multifractal spectrum* (MFS) is defined as a relation between local and outer dimension:

(4b) $$\begin{array}{c} D_{M}\left(d_{\mu }\right)\equiv D_{M}\left(F_{i}\right). \end{array}$$

Dimension *D* is defined with respect to measure *M* while local dimension *d* is defined with respect to local measure *μ*. In fact, outer dimension gives information on complexity of each monofractal subset. While local dimension seeks for local singularities and trends, outer dimension is calculated on the whole set of points with the same value of the local dimension. Usually, *D* is Hausdorff dimension when MFS is denoted as a *fine MFS*, whilst *d* is calculated using arbitrary local measure or capacity. Multifractal spectrum is usually in the literature denoted as *f*(*α*) [2].

## 3. Fractal Modeling in Image Processing

There are two approaches of fractality in image processing. The first approach [3, 16–18] assumes image as a surface with pixels represented as triplets (*x*, *y*, *z*) where *x* and *y* are spatial coordinates and *z* is pixel intensity. Thus, pixel intensity is treated as two spatial coordinates. In turn this involves embedded dimension to be 3 and treats an image geometrically. Second approach [14] models pixels as triplets (*x*, *y*, *f*(*x*, *y*)) where *x* and *y* are again spatial coordinates while *f*(*x*, *y*) is pixel intensity and it is modeled as a function over spatial coordinates. An image is seen as an object embedded in 2D space with strict distinction between spatial and pixel intensity components. This model is closer to traditional image processing techniques.

Both approaches address model described in the previous section: in the first approach the dimension is calculated for the 3D object from the geometrical viewpoint via area measuring and calculations are done in 3D space, while in the second case the dimension is calculated for the function *f*(*x*, *y*) that models pixel intensities in the 2D space and *μ*_*H*_(*f*(*F*_*i*_), *s*) is calculated.

## 4. Blanket Method

Here we shall give a brief overview of blanket method proposed for calculating dimension of a monofractal set of points [3]. Blanket method represents an example of the first (3D embedded space) approach from previous section. The main idea of this approach of calculating fractal dimension of unidimensional set of points is to employ area of the image as a global measure. Area is computed indirectly via volume, that is, blanket constructed around original image surface through successive iterations of thickening of the blanket. Upper, *u*_*ε*_(*x*, *y*), and lower surface, *b*_*ε*_(*x*, *y*), of the blanket in *ε*th iteration, respectively, are given by

(5a) $$\begin{array}{c} u_{\varepsilon }\left(x,y\right)=\max\left\{u_{\varepsilon -1}\left(x,y\right)+1,\underset{S_{1}\left(x,y\right)}{\max}u_{\varepsilon -1}\left(x,y\right)\right\}, \end{array}$$

(5b) $$\begin{array}{c} b_{\varepsilon }\left(x,y\right)=\min\left\{b_{\varepsilon -1}\left(x,y\right)-1,\underset{S_{1}\left(x,y\right)}{\min}b_{\varepsilon -1}\left(x,y\right)\right\}, \end{array}$$

(5c) $$\begin{array}{c} u_{0}\left(x,y\right)=b_{0}\left(x,y\right)=f\left(x,y\right). \end{array}$$

In these equations *S*_1_(*x*, *y*) represents 3 × 3 pixel neighborhood and *f*(*x*, *y*) denotes pixel intensity. The first term in the brackets (addition or subtraction of 1) ensures thickening of the blanket in every iteration. Volume of the blanket is calculated as a sum of differences:

(5d) $$\begin{array}{c} V_{\varepsilon }\left(F_{\varepsilon }\right)=\sum_{F_{\varepsilon }}\left[u_{\varepsilon }\left(x,y\right)-b_{\varepsilon }\left(x,y\right)\right]. \end{array}$$

Finally, measure (area) is given by dividing volume by minimal thickness of the blanket, *ε*:

(5e) $$\begin{array}{c} M_{\text{BLANKET}}\left(F_{\varepsilon }\right)=\frac{V_{\varepsilon }}{2\varepsilon }. \end{array}$$

Although the gauge in this method is of dimension 3 (blanket is three dimensional object), the final measure is of dimension 2, since the area is calculated ultimately. If we recall here the MF principles, then thickness of the blanket corresponds to scale and object is regarded as a monofractal a priori.

## 5. Multifractal Blanket Method

### 5.1. Previous Work

Blanket method is widely used in image processing through many different approaches. In [19, 20] image is divided in blocks (subimages) and FD is calculated for each block by direct implementation of blanket method. Local fractal dimension (LFD) for blanket method is proposed in [21]. Here, locality considers the gliding window of fixed size on which the classical blanket method is implemented for each pixel, that is, only blanket thickness is scaling. Obtained matrices are called *LFD maps*. LFD concept is generalized for volumetric textures in [22]. Further multifractal (global fractal) analysis for LFD is not provided in these papers.

LFD notion implies pixel-wise implementation of so-called global fractal dimension, that is, classical blanket method described in previous section. This concept is quasimultifractal approach.

Concerning multifractal approach, it is presented in the literature mainly through use of coarse multifractal spectrum [23], especially Legendre MFS (see the Appendix). Calculation of fractal dimension is mainly based on box-counting (BC) capacity and its modifications, for example, differential box-counting (DBC) capacity. All these capacities assume an image embedded in 3D space. Fine MFS is used only when image is modeled as a 2D object [9, 24]. In this section, new local multifractal measures of both types are proposed and are inspired by classical blanket method.

### 5.2. Proposed 3D Multifractal Framework (Method 1)

Instead of a pixel-wise implementation of blanket method, here is proposed a local measure based on blanket method which can be used for calculation of local dimension in a sense of fine MF formalism described in (3).

In classical blanket method upper and lower surfaces (5a), (5b), alternatively, can be rewritten as follows:

(6a) $$\begin{array}{c} u_{\varepsilon }\left(x,y\right)=\max\left\{u_{0}\left(x,y\right)+\varepsilon ,\underset{S_{\varepsilon }\left(x,y\right)}{\max}u_{0}\left(x,y\right)\right\}, \end{array}$$

(6b) $$\begin{array}{c} b_{\varepsilon }\left(x,y\right)=\min\left\{u_{0}\left(x,y\right)-\varepsilon ,\underset{S_{\varepsilon }\left(x,y\right)}{\min}u_{0}\left(x,y\right)\right\}. \end{array}$$

This equation gives a possibility of defining a local measure inspired by blanket method. Hence, the local volume is computed over an *ε*—neighborhood (*ε* × *ε* pixels neighborhood) of each pixel, *S*_*ε*_(*x*, *y*), and it represents local blanket measure:

(6c) $$\begin{array}{c} \mu_{\text{BLANKET}}\left({ℬ}_{\varepsilon }\left(x\right)\right)=\sum_{S_{\varepsilon }\left(x,y\right)}\left[u_{\varepsilon }\left(x,y\right)-b_{\varepsilon }\left(x,y\right)\right]. \end{array}$$

It is worth saying that not only the thickness of the blanket is scaled but also simultaneously the pixel neighborhood is scaled according to MF formalism.

### 5.3. Proposed 2D Multifractal Framework (Method 2)

Measure presented in (6a), (6b), and (6c) assumes an image as a surface embedded in 3D space, since measure considers intensities interchangeably with spatial coordinates. In order to create a measure for model of an image where the pixel intensities are seen as a function over spatial coordinates, equations in (6a) and (6b) are revisited as

(7a) $$\begin{array}{c} u_{\varepsilon }\left(x,y\right)=\max\left\{w_{1}\cdot u_{0}\left(x,y\right),\underset{S_{\varepsilon }\left(x,y\right)}{\max}u_{0}\left(x,y\right)\right\}, \end{array}$$

(7b) $$\begin{array}{c} b_{\varepsilon }\left(x,y\right)=\min\left\{w_{2}\cdot u_{0}\left(x,y\right),\underset{S_{\varepsilon }\left(x,y\right)}{\min}u_{0}\left(x,y\right)\right\}, \end{array}$$

where *w*_1_ and *w*_2_ are weights which simulate the expansion of the blanket in (6a), (6b), and (6c). Equations in (7a) and (7b) are not depending on blanket thickness. Local dimension can be computed using (6c) and the fine MFS as defined in (4a) and (4b).

Further inspection of this measure indicates its correlation with three well known capacities: MIN, MAX, and SUM [9]. It is indeed superposition of these three capacities with correction term causing monotony of the measure.

### 5.4. Blanket Multifractal Spectrum (Method 3)

Classical blanket method considers an image as a monofractal object. Otherwise if multifractality is taken into consideration, there should be used decomposition of the image on monofractal partitions. Local measure can be chosen arbitrary. For each of the partitions blanket measure (5a), (5b), (5c), (5d), and (5e) and blanket dimension are then calculated. Obtained spectrum can be seen as the *blanket multifractal spectrum*. The only restriction is to use local measure based on approach where pixel intensity is modelled as a third spatial coordinate.

## 6. Experimental Results

Mathematical model of multifractality given in (4a) and (4b) assumes infinitesimal range of scales. There should be made a difference between original data (infinitesimally increasing resolution of a view of a scene) and its sampled version (digital image) [5]. Image resolution is finite and some numerical assumptions should be considered. Therefore, windows and box sizes (or scales) used in calculation of local and global dimension, respectively, of the image are discussed in this section in detail. Performed analysis is focused primarily on discrimination potential of proposed MF spectra.

This section ends with examination of appropriateness of proposed methods for calculation of MF spectra to discriminate different textures. In applications dimension and MFS are used as signatures of images and implemented for discrimination. Fractal modelling in texture classification is commonly implemented through calculation of fractal dimension (FD), where each texture is described by single value (FD value). Since two different textures can be of the same dimensionality, one more value—lacunarity [1, 25, 26]—is usually employed. Some authors [1, 27] propose use of succolarity for better discrimination. Multifractal spectrum is often used as a discriminator [9, 10].

### 6.1. Least Squares Approximation

Calculation of dimension, regardless of local or global dimension, is based on power law. This is clear from (1a) and (1b), (2a) and (2b), and (3) and it can be in general written as

(8a) $$\begin{array}{c} \text{measure}=C\cdot \text{scal}{\text{e}}^{\text{dimension}} \end{array}$$

or after taking logarithm on both sides as

(8b) $$\begin{array}{c} \log\left(\text{measure}\right)=\log\left(C\right)+\text{dimension}\cdot \log\left(\text{scale}\right). \end{array}$$

Calculation of dimension in this model implies determining measure variations while varying scale. Our model from Section 2 assumes dimension and *C* to be constants. In order to facilitate writings, substitution of variables provides

(8c) $$\begin{array}{c} \hat{y}=k\cdot x+n, \end{array}$$

where $\hat{y}=\log\left(\text{measure}\right)$, *k* = dimension, *x* = log⁡⁡(scale), and *n* = log⁡⁡(scale). Therefore, our model in (8c) predicts values $\hat{y_{i}}$ while measured values (of logarithm of measure) are *y*_*i*_. Determination of value *k* (i.e., dimension) in fractal modeling is usually done through minimisation of *least squares error *[28] defined as

(9) $$\begin{array}{c} e\equiv \sum_{i=1}^{N}{\left\{y_{i}-\hat{y_{i}}\right\}}^{2}, \end{array}$$

where *N* is the total number of scales. Differentiating least squares error both for *k* and *n* and putting derivatives to zero (∂*e*/∂*k* = 0, ∂*e*/∂*n* = 0) gives a dimension value

(10) $$\begin{array}{c} k=\frac{N\sum_{i=1}^{N}x_{i}y_{i}-\left\{\sum_{i=1}^{N}x_{i}\right\}\left\{\sum_{i=1}^{N}y_{i}\right\}}{N\sum_{i=1}^{N}{x_{i}}^{2}-{\left\{\sum_{i=1}^{N}x_{i}\right\}}^{2}}. \end{array}$$

### 6.2. Multifractal Spectrum Calculation

In this section all MF spectra are calculated using *histogram method* [2, 28, 29] and box-counting (BC) capacity (see the Appendix). Actually, here numerical evaluation of equations given in (4a) and (4b) is shortly described. After determining local dimension values, *d*_*i*_, using least squares approximation, there is a range of local dimension values, *d* ∈ (*d*_min⁡_, *d*_max⁡_). According to (4a) and (4b) regions in image with the same value of local dimension should be exploited for calculation of global dimension. Straightforward application of that principle is unacceptable and some rounding must be done. The easiest solution is to uniformly quantize local dimension range to arbitrary resolution (number of uniformly distant levels), *R*. For each region with the same local dimension value, global fractal dimension is then calculated.

### 6.3. Multifractal Spectra Inspection

**Figure 3:**
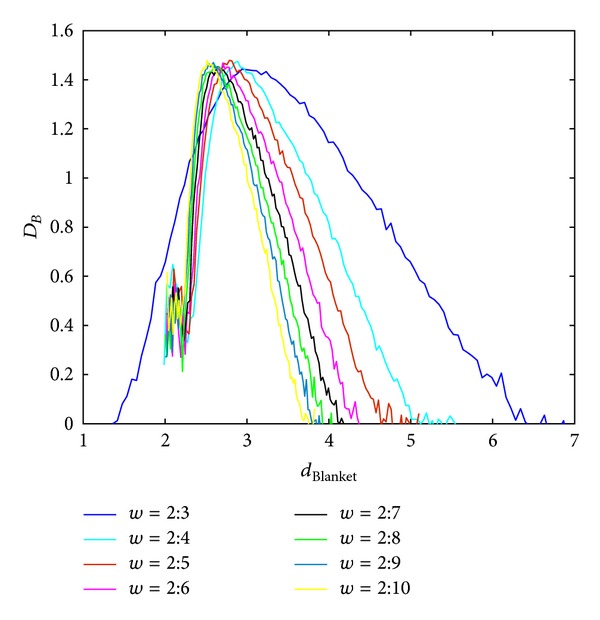
BC MFS obtained using Method 1 for different window sizes, *w*, for calculation of local dimension. Notation *A* : *B* implies set of values *A*, *A* + 1,…, *B*.

**Figure 4:**
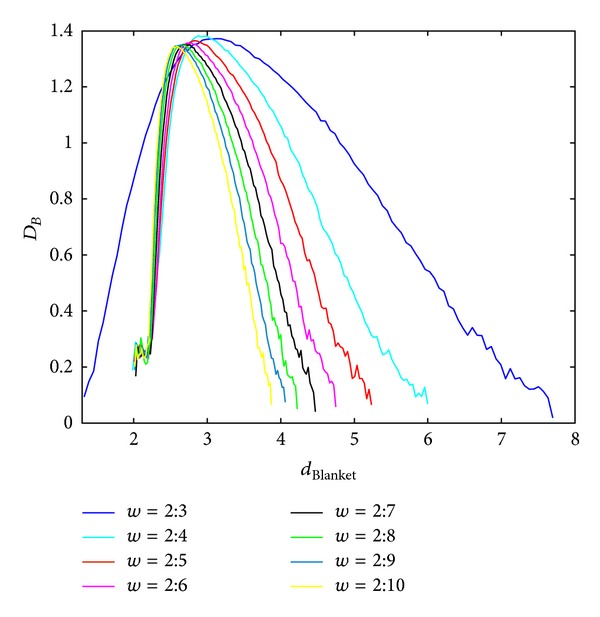
BC MFS obtained using Method 2 for different windows sizes, *w*, for calculation of local dimension. Notation *A* : *B* implies set of values *A*, *A* + 1,…, *B*.

Local dimension considers local behaviour of the points (pixels) and consequently used rectangular windows are supposed to be lower. Behaviour of the BC MF spectrum with respect to different windows sizes in calculating local dimension is shown in Figures 3 and 4, for Method 1 and Method 2, respectively. Image analysed in this section is of size 640 × 640 pixels. Parameters for calculating MFS are the same for each curve, while only scales for local dimension calculation are varying. From Figures 3 and 4 it is evident MFS shrinkage while the form of the MFS does not change significantly.

**Figure 5:**
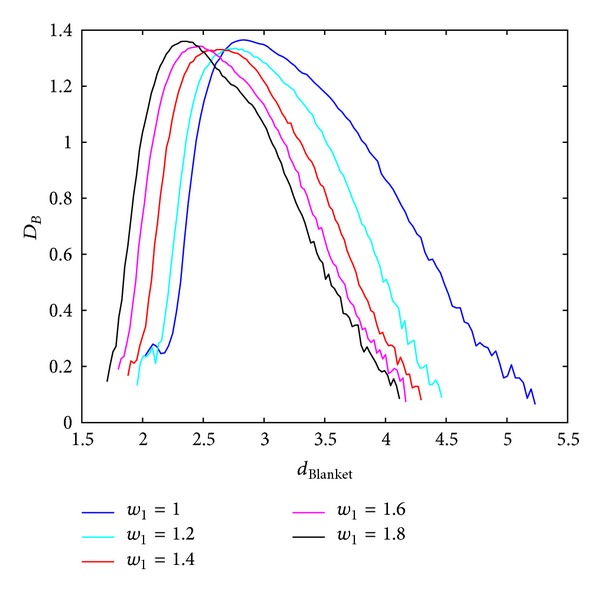
BC MFS obtained using Method 2 for different values of weight *w*_1_.

**Figure 6:**
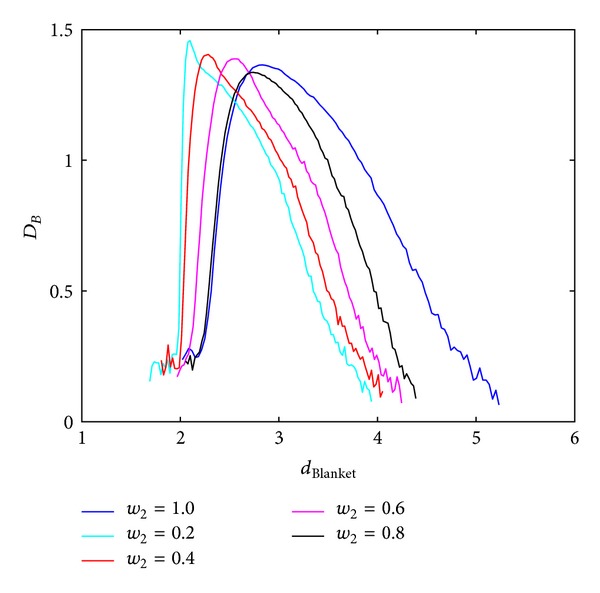
BC MFS obtained using Method 2 for different values of weight *w*_2_.

Weights *w*_1_ and *w*_2_ in proposed Method 2 and their influence on BC MFS are analyzed in Figures 5 and 6. Parameter *w*_1_ is always greater than 1 while parameter *w*_2_ is smaller than 1. The reason for this is in providing monotony as explained in Section 5. Hence, parameter *w*_1_ avoids upper blanket to be lower in every successive iteration. The same stands for parameter *w*_2_ and lower blanket.

**Figure 7:**
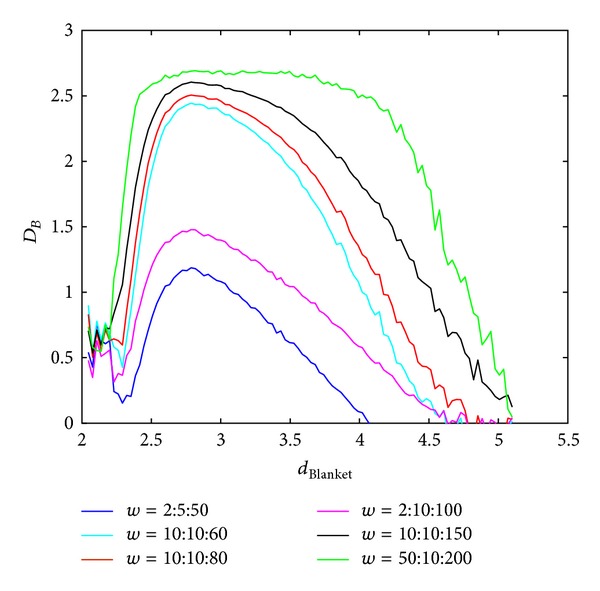
BC MFS obtained using Method 1 for different box sizes, *w*, for calculation of global dimension. Notation *A* : *B* : *C* implies set of values *A*, *A* + *B*,…, *C*.

**Figure 8:**
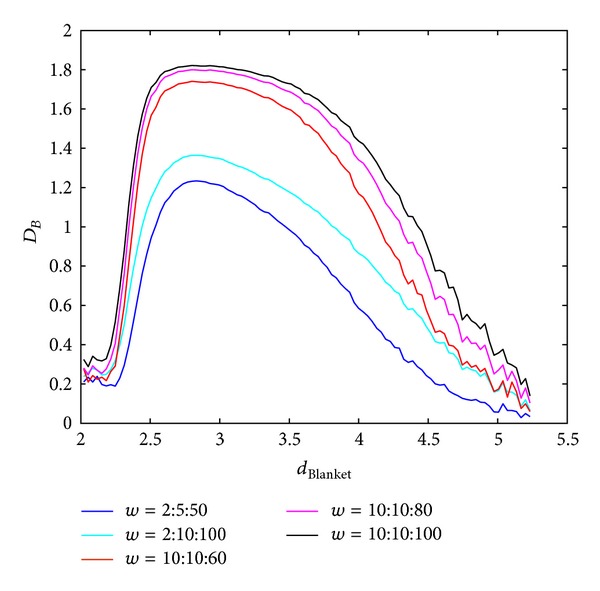
BC MFS obtained using Method 2 for different box sizes, *w*, for calculation of global dimension. Notation *A* : *B* : *C* implies set of values *A*, *A* + *B*,…, *C*.

The influence of chosen box sizes on calculation of BC MF spectra in Method 1 and Method 2 is shown in Figures 7 and 8. Higher ranges of window sizes produce spectra which are not discriminative regarding wide range of local dimension values. On the other hand small windows disregard global behaviour of the image and accent only local singularities.

**Figure 9:**
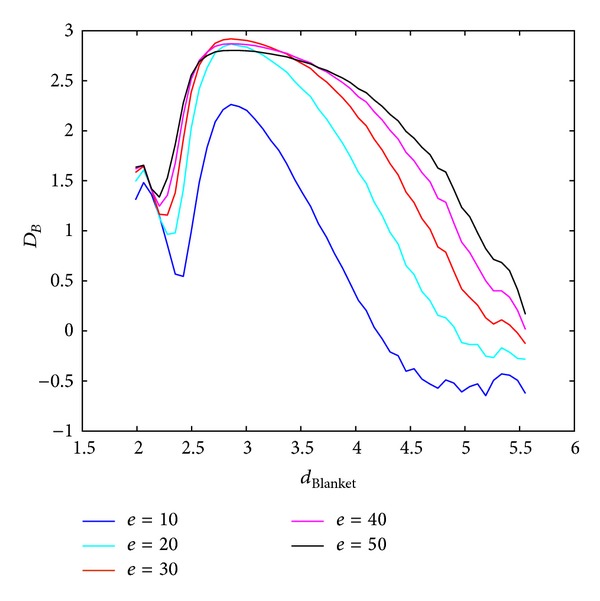
Blanket MFS (Method 3) obtained for different blankets, *e*.

Finally, MF blanket spectrum is analyzed for different blankets (Figure 9). After blanket of size 30, higher values of blanket do not induce significant changes in the shape of MFS and there is evident rounding of MFS curve.

### 6.4. Texture Discrimination

**Figure 10:**
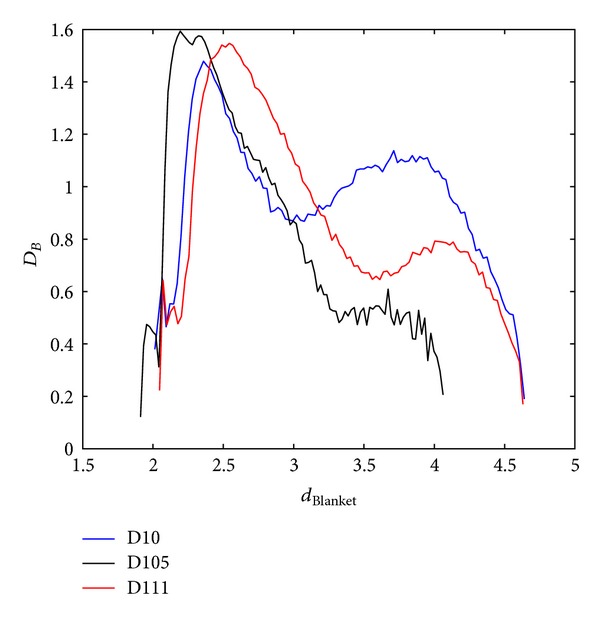
BC MFS obtained with Method 1 for three different textures from [30].

**Figure 11:**
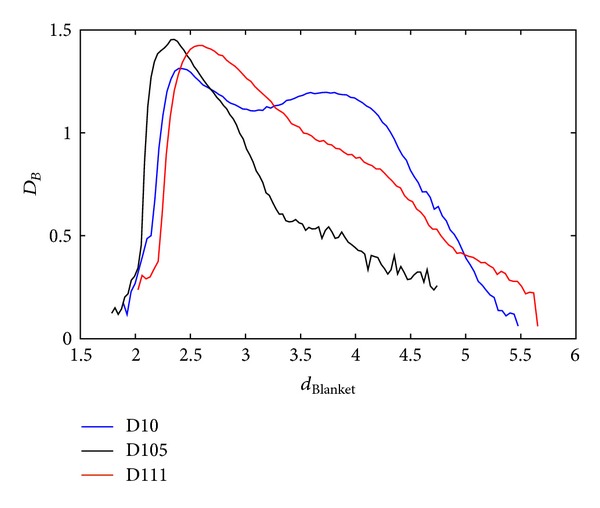
BC MFS obtained with Method 2 for three different textures from [30].

**Figure 12:**
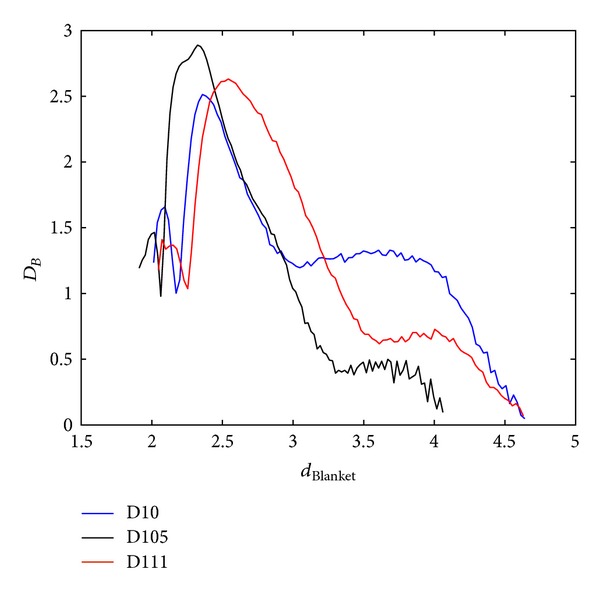
Blanket MFS for three different textures from [30]. Local dimension values are calculated using local MF measure from Method 1.

Each dimension and measure describes particular singularity and this section explores potentials of proposed measures. As an illustration of MF spectra diversity, spectra of three random textures from [30] are calculated and shown in Figures 10, 11, and 12, for all three proposed methods. When calculating MF according to Method 1 and Method 2, BC method is used for calculation of global dimension. Method 3 assumes local dimension calculation from Method 1.

**Figure 13:**
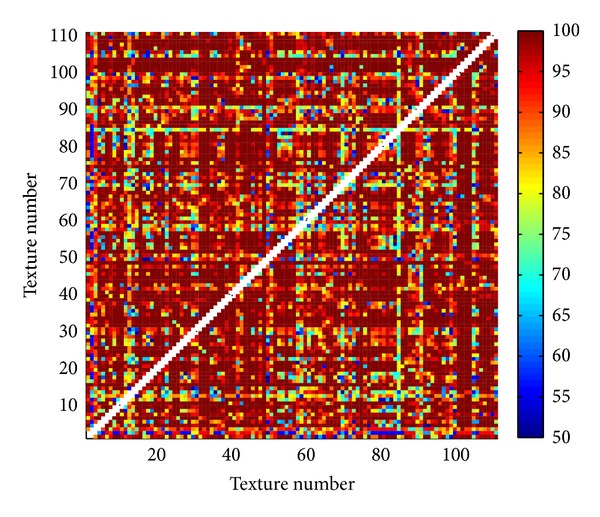
Percentage of correctly classified pairs of textures from the Brodatz database for Method 1. Texture order corresponds to [31].

**Figure 14:**
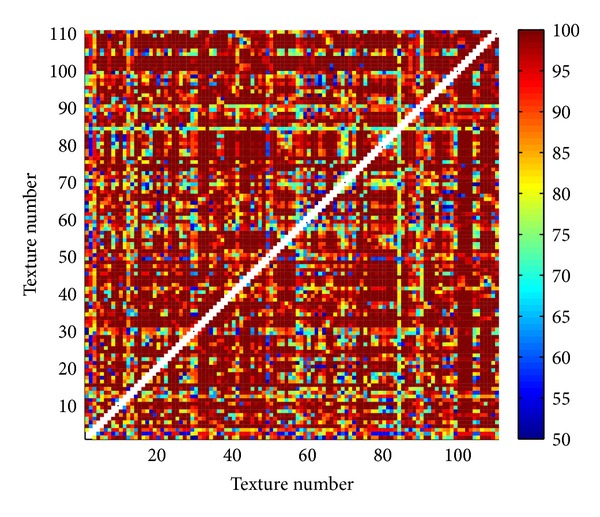
Percentage of correctly classified pairs of textures from the Brodatz database for Method 2. Texture order corresponds to [31].

**Figure 15:**
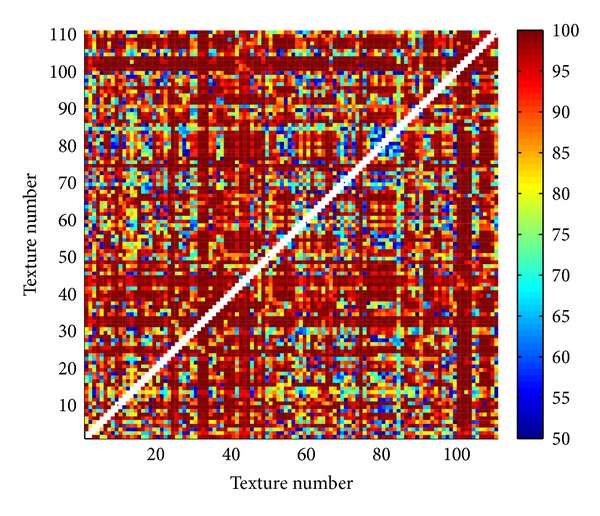
Percentage of correctly classified pairs of textures from the Brodatz database for Method 3. Texture order corresponds to [31].

Proposed three methods are tested on Brodatz database [30, 31] with 111 texture images. Each texture (640 × 640 pixels) is divided into 25 images (128 × 128 pixels). Within obtained database of totally 2775 images two different textures (25 images of one and 25 of the other texture) are classified. For classification K-means method [32] is used. Since this method highly depends on initial cluster centroid positions, classification is repeated for 400 times. As a result of classification the solution is used with the lowest sum of distances to every centroid. Correctness rate is calculated as a ratio between correctly classified images within both textures and the total number of images (50). Results of classification for all pairs of textures in the database are illustrated in Figures 13, 14, and 15. In matrices in these figures color of each point corresponds to the percent of correctly classified images belonging to one of two Brodatz textures defined by abscissa and ordinate values.  Table 1 brings mean correctness rate for all three methods with variation of some parameters in calculation of MFS. For Methods 1 and 2 both sizes of boxes (MFS scales) and resolution of MF spectrum vary. Average computation time of features in Method 3 is the main reason why only one test is done. As it is obvious from Table 1 obtained precision is even inferior to the other two methods. Computations are done on Intel Core i5 3 GHz processor.

Comparing results of classification between different methods (Table 1), Methods 1 and 2 are superior regarding both correctness rate and average computation time. Classification is performed also with classical blanket method [3] and obtained mean correctness is 84.5281% (maximum blanket thickness is 20). From Figures 13, 14, and 15 it is evident that misclassifications occur almost for the same pairs of textures in all three scenarios. One of the reasons for lower classification rates is high variation between images within the same class. If the texture pattern is large then images obtained by division of this texture will substantially differ.

## 7. Conclusion

Proposed multifractal measures correspond to two models in fractal modeling of images. To our knowledge measure introduced within Method 1 is the first local measure assuming pixel intensity as a third spatial coordinate whose fine MFS is calculated. Usually only Legendre MFS is calculated for such measures. Rearranging this measure into measure with pixel intensity modeled as a function over spatial coordinates transposes Method 1 to Method 2 through dimension reduction. Conducted tests suggest nearly identical results according to both precision and time consumption. Further reduction of computation time could be done by calculation of MFS only for a region of a texture, since there is a repetition of texture's pattern within the texture. As results show this must be done empirically for each texture because of the ratio between sizes of the pattern and the whole texture.

## Figures and Tables

**Figure 16:**
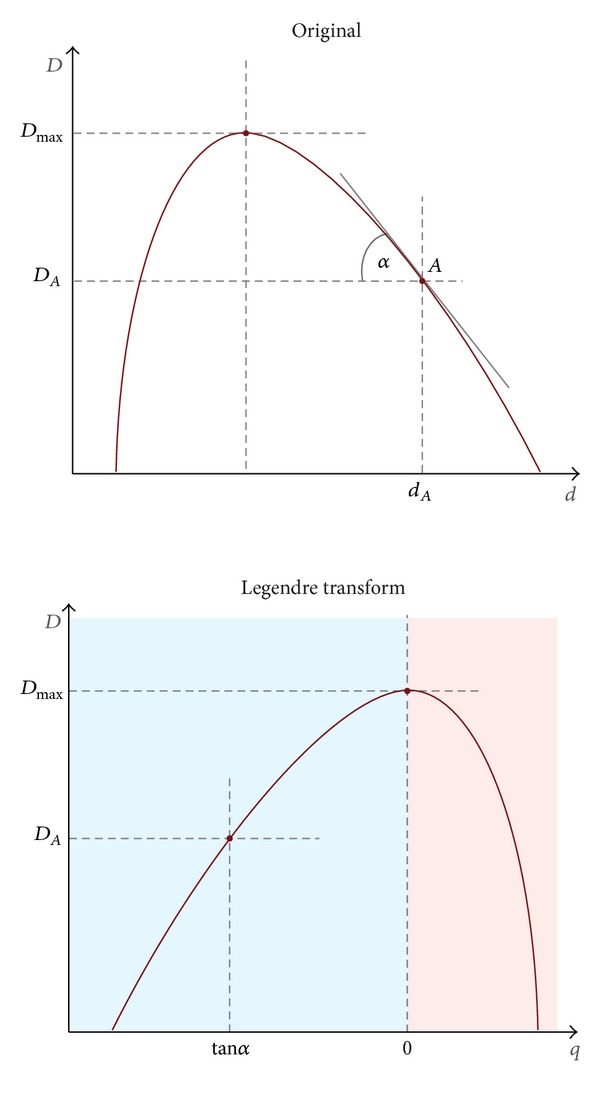
Legendre transform illustration. Red and blue domains evoke regions with positive or negative values for *q*. Here Legendre transform curve is a reflected image of original function, but it is not true in general.

**Table 1:** Comparison of proposed three methods regarding precision and computation time.

Method	MFS scales	MFS resolution	Mean correctness (%)	Average time per pixel (*d* values) (µs/pix)	Average time per pixel (*D* values) (µs/pix)
1	2 : 10 : 72	20	92.2234		1.8443
		20	93.7808	15.5874	1.9072
	2 : 2 : 20	15	93.1751		1.4749
		10	93.5119		1.0510

2	2 : 10 : 72	20	90.9808		3.2328
		20	92.4177	15.2868	3.4039
	2 : 2 : 20	15	92.1202		2.6124
		10	91.5135		1.8256

3	7	20	88.4986	15.5874	39.0058

All experiments are performed on processor Intel Core i5, 3 GHz.

Notation in second column *A* : *B* : *C* indicates values *A*, *A* + *B*, *A* + 2*B*,…, *C*.

All *d* values were calculated using window sizes 2, 3, 4, and 5. In Method 2 parameters *w*_1_ and *w*_2_ are equal to one.

## Appendix

This appendix brings simple and efficient technique for estimating Hausdorff MFS via Legendre transform with aim to comprehend MFS calculation context. In the literature it is usually but mistakenly introduced as MF formalism already described by (3) and (4a) and (4b). Legendre MFS has its origin in statistical mechanics and terminology used in derivation of this spectrum is taken from there.

Hausdorff measure is given by

(A.1) $$\begin{array}{c} M_{H}\left(F_{i},s\right)=\underset{\delta \to 0}{\lim}\inf\sum_{\begin{array}{c} j=1\\ F_{i} \end{array}}^{\infty }{\left|U_{j}\right|}^{s}. \end{array}$$

Since *δ*-covering is hardly realizable in practice, when there is no knowledge of fractal object synthesis (creation), it is reasonable to tile the object with covers of the same length, *δ*:

(A.2) $$\begin{array}{c} M_{B}\left(F_{i},s\right)=\underset{\delta \to 0}{\lim}\inf\sum_{F_{i}}\delta^{s}. \end{array}$$

*M*_*B*_ is called *box-counting capacity* and dimension resulting from it is called *box-counting dimension*, *D*_*B*_. Box-counting dimension is an overestimation of Hausdorff dimension [2]:

(A.3) $$\begin{array}{c} D_{H}\left(F_{i}\right)\leq D_{B}\left(F_{i}\right). \end{array}$$

Box-counting capacity leads to so-called *coarse MFS*. It is often desirable to calculate MFS in specific points such as a maximum of MFS function or in a point with *D* = *d*. Shapes of MF spectra of different objects (images) can be compared in space of particular points. With this motivation let us transform function *D*_*B*_ of variable *d*_*μ*_ into another variable, which represents first derivative of dimension, *q* = *dD*_*B*_/*dd*_*μ*_. Legendre transform provides it:

(A.4) $$\begin{array}{c} {\left(D_{B}\left(d_{\mu }\right)\right)}^{*}=D_{B}^{*}\left(q\right)=\underset{d_{\mu }}{\inf}\left\{q\cdot d_{\mu }-D_{B}\left(d_{\mu }\right)\right\}. \end{array}$$

Alternatively Legendre transform can be defined using supremum (maximum) instead of infimum (minimum) [33].

Transformation given with (*D*_*B*_, *D*_*B*_*) is Legendre transformation pair, hence, the name of the corresponding spectrum.  Figure 16 shows how Legendre transform centers maximum of spectrum into zero in space of *q*-variable. If *D*_*B*_ is concave, that is, ∂^2^ *D*_*B*_/∂*d*_*μ*_^2^ ≤ 0, then (A.4) is valid without brackets. Legendre transform is *involutive* [34], that is, Legendre transform of *D*_*B*_* is *D*_*B*_. In other words direct and inverse Legendre transforms are identical. Substitution of transformed dimension gives the following capacity:

(A.5) $$\begin{array}{c} M_{L}\left(F_{i},D_{B}\right)=\delta^{-D_{B}^{*}}\sum_{F_{i}}\delta^{q\cdot d_{\mu }}=\delta^{-D_{B}^{*}}\sum_{F_{i}}\mu {\left({ℬ}_{\delta }\left(x\right)\right)}^{q},\\ M_{L}\left(F_{i},D_{B}\right)\leq \delta^{-D_{B}^{*}}\sum_{F}\mu {\left({ℬ}_{\delta }\left(x\right)\right)}^{q}. \end{array}$$

The term on the right side of inequality, that is, sum of moments, stems for *partition function *[35], Γ(*q*) = ∑_*F*_ *μ*(*ℬ*_*δ*_(**x**))^*q*^, from the analogy with thermodynamics. It depends only on *q* but not on scale, *δ*. Thus, Legendre transform of dimension is

(A.6) $$\begin{array}{c} D_{B}^{*}\left(q\right)\sim \frac{\log\sum_{F}\mu {\left({ℬ}_{\delta }\left(x\right)\right)}^{q}}{-\log\delta }. \end{array}$$

This variable is often labeled in literature as −*β*(*q*) [2] or *τ*(*q*) [35]. Applying chosen measure *μ* on blocks within the mesh grid of resolution *δ* and calculating *q*th power moment sums of *μ* gives the value of Legendre transform for each *q*. Legendre transform of *D*_*B*_*(*q*) gives MFS, *D*_*B*_. Local dimension values, needed for calculation of *D*_*B*_, are also determined via *D*_*B*_* by equating ∂*D*_*B*_/∂*q* = 0:

(A.7) $$\begin{array}{c} d_{\mu }=\frac{\partial D_{B}^{*}}{\partial q}, \end{array}$$

therefore,

(A.8) $$\begin{array}{c} D_{B}=q\cdot \frac{\partial D_{B}^{*}}{\partial q}-D_{B}^{*}. \end{array}$$

As it is obvious from the derivation of Legendre MFS, this numerical method provides only pairs in the graph *D*_*B*_(*d*_*μ*_), without calculating local and global dimension values for each point of the signal (function). This method executes faster on computer although the precision is sacrificed.

Legendre MFS can be calculated not only by box-counting method but also by using gliding-box method [36]. This method calculates partition function on slipping box, that is, for every pixel. Reported results are better than using box-counting calculation.
